# Performance of Cystatin C-Based Equations for Estimation of Glomerular Filtration Rate in Diabetes Patients: A Prisma-Compliant Systematic Review and Meta-Analysis

**DOI:** 10.1038/s41598-018-38286-9

**Published:** 2019-02-05

**Authors:** Amanda Veiga Cheuiche, Marina Queiroz, André Luis Ferreira Azeredo-da-Silva, Sandra Pinho Silveiro

**Affiliations:** 1 Graduate Program in Medical Science: Endocrinology, Universidade Federal do Rio Grande do Sul (UFRGS), Porto Alegre, Brazil; 2 Internal Medicine Division, Hospital de Clínicas de Porto Alegre (HCPA), Porto Alegre, Brazil; 3 Endocrine Division, HCPA, Porto Alegre, Brazil

## Abstract

The accuracy of estimated glomerular filtration rate (eGFR) equations in diabetes mellitus (DM) patients has been extensively questioned. We evaluated the performance of cystatin C-based equations alone or in combination with creatinine to estimate GFR in DM patients. A PRISMA-compliant systematic review was performed in the MEDLINE and Embase databases, with “diabetes mellitus” and “cystatin C” as search terms. Studies comparing cystatin C-based eGFR equations with measured GFR (mGFR) in DM patients were eligible. Accuracies P10, P15, P20, and P30 indicated the proportion of eGFR results within 10, 15, 20, and 30% of mGFR. Single-arm meta-analyses were conducted, and the Quality of Diagnostic Accuracy Studies-II tool (QUADAS-2) was applied. Twenty-three studies comprising 7065 participants were included, and 24 equations were analyzed in a broad range of GFRs. Meta-analyses were completed for 10 equations. The mean P30 accuracies of the equations ranged from 41% to 87%, with the highest values found with both CKD-EPI equations. Mean P10-P15 achieved 35% in the best scenario. A sensitivity analysis to evaluate different mGFR methods did not change results. In conclusion, cystatin C-based eGFR equations represent measured GFR fairly at best in DM patients, with high variability among the several proposed equations.

## Introduction

According to the 2017 United States Renal Data System (USRDS) Annual Data Report, 40% of individuals living with chronic kidney disease (CKD) in the United States also had diabetes mellitus (DM)^1^. These data are consistent with the results of the Brazilian Longitudinal Study of Adult Health (ELSA)-Brazil multicenter cohort survey, in which 42% of people with CKD had concomitant diabetes^2^.

As for more advanced stages, the 2015 European Renal Association − European Dialysis and Transplant Association (ERA-EDTA) Registry Annual Report found that, among patients starting renal replacement therapy, one-quarter had DM as their primary renal diagnosis^3^. A recent systematic review demonstrated substantial variation in the incidence of end-stage renal disease (ESRD) among patients with diabetes, but a consistent finding was the excess risk of advanced kidney disease among this population when compared to non-diabetic individuals, ranging from 6- to 60-fold higher in diabetes^4^.

To detect diabetic kidney disease (DKD), up-to-date guidelines recommend annual screening with measurement of urinary albumin excretion (UAE) in a spot urine sample and estimation of glomerular filtration rate (eGFR) with creatinine and/or cystatin C-based equations^5^. Specific use of serum cystatin C-based equations is recommended when creatinine-based equations yield questionable results, such as for patients with creatinine-based eGFR values between 45 and 60 mL/min/1.73 m^2^ and no other markers of kidney damage^6^.

The use of cystatin C-based equations is also favored in situations that affect serum creatinine levels, such as in patients with muscle mass loss secondary to limb amputations or neurological diseases^7^. In the DM population, it has been demonstrated that creatinine-based equations may markedly underestimate GFR, especially in patients with GFRs within normal or elevated ranges^8–10^. This worse performance seems to be related to analytical interference of hyperglycemia on creatinine measurement and decreased sensitivity of creatinine to identify glomerular hyperfiltration^8–11^. In this context, there has been increasing interest in validating alternative markers. One such marker is cystatin C, an endogenous protein produced at a constant rate by nuclear cells, that is freely filtered by the glomerular membrane, being neither reabsorbed nor secreted in the kidney tubular system.

Several cystatin C-based equations have been developed for use in different populations. In patients with DM, a subset of studies has demonstrated superiority of these equations when compared to creatinine-based ones^12,13^. However, other studies disagree, demonstrating similar or even worse performance of cystatin C-based equations^14–16^. A recent proposal suggested the combined use of cystatin C- and creatinine-based equations, which seems to increase accuracy when compared to the use of either indicator alone^17–19^.

Within this context, the present study was designed to evaluate the accuracy of serum cystatin C-based equations, either alone or combined with serum creatinine, to estimate GFR in DM patients with a wide spectrum of renal function, by means of a systematic review of the literature and meta-analysis.

## Methods

### Protocol and registration

This systematic review and meta-analysis follows the recommendations of the Preferred Reporting Items for Systematic Reviews and Meta-analyses (PRISMA) protocol^20^, and was registered in the International Prospective Register of Systematic Reviews (PROSPERO) with accession number CRD42018033197.

### Eligibility criteria

We included studies that compared the accuracy of serum cystatin C-based GFR-estimating equations to represent GFR measured (mGFR) by reference methods (^125^I-iothalamate, ^51^Cr-EDTA, ^99m^Tc-DTPA, inulin, iohexol) in patients with diabetes. Studies that evaluated patients with diabetes as a subgroup were included if there was available information.

The exclusion criteria were absence of data on the performance of the equation evaluated, animal studies, and review studies. There was no language or date restriction; articles written in languages other than English, Portuguese, and Spanish were considered eligible if they contained sufficient English-language information in the abstract, tables, and figures.

### Search strategy and study selection

We conducted a systematic search of the MEDLINE (via PubMed) and Embase databases, from inception to October 2017. Comprehensive search queries included descriptors (MeSH and Emtree) based on the expressions “diabetes mellitus” and “cystatin C” (index test). The complete search strategy is provided as a supplement.

Two independent reviewers (A.V.C. and M.Q.) assessed records for inclusion based on titles and abstracts. Abstracts that did not meet the inclusion criteria or that met the exclusion criteria were discarded. The remaining records and those whose abstracts did not provide sufficient information to decide upon their exclusion were selected for full-text evaluation, which was performed by the same reviewers independently. A third reviewer (S.P.S.) solved disagreements.

### Data collection and extraction

Two investigators (A.V.C. and M.Q.) analyzed the selected studies and extracted data using a standardized system. The following information were obtained: first author, year of publication, study design, sample size, age distribution, mean mGFR, type of diabetes, cystatin C equation, reference method for mGFR, method of cystatin C measurement and whether it was traceable to the reference method, accuracy and correlation of the equation with measured GFR. All included equations are described in the supplement.

### Risk of bias in individual studies and quality of meta-analysis

The risk of bias and the quality of studies were assessed with the Quality of Diagnostic Accuracy Studies-2 (QUADAS-2) tool^21^.

### Diagnostic accuracy measures

Accuracy was defined as the percentage of GFR estimates within 10% (P10), 15% (P15), 20% (P20), 30% (P30), and 50% (P50) of mGFR. The coefficient of correlation between eGFR and mGFR was recorded.

### Synthesis of results and meta-analysis

The number of patients whose eGFR was within P10, P15, P20, and P30 of mGFR was entered into a single arm meta-analysis, carried out in CMA software version 3.0 to obtain an overall estimate of accuracy for each set of studies that used the same equation. We used the random effects model to incorporate heterogeneity, estimated by the inconsistency test (I^2^) proposed by Higgins *et al*.^22^. Cutoff points of 25 and 75% were used to classify heterogeneity as low, moderate, or high.

### Additional analyses

In cases of moderate or high heterogeneity, we performed sensitivity analyses to evaluate the effect of different reference methods for mGFR.

## Results

**Figure 1:**
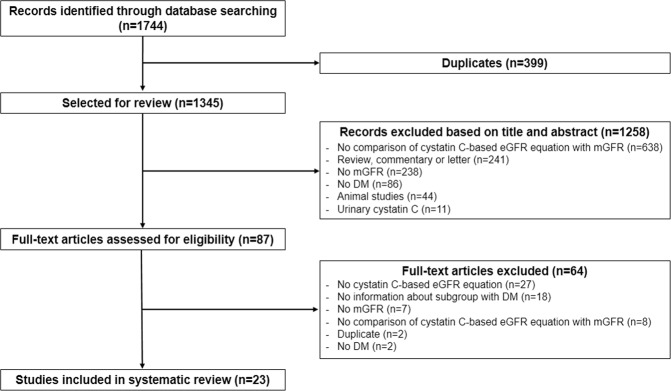
Study flowchart.

The search strategy identified 1744 citations, of which 1345 were left after removal of duplicates. We excluded 1258 articles after title and abstract screening, leaving 87 studies for full-text evaluation. Finally, 23 studies including 7065 participants, with a broad range of GFR values, were selected for the systematic review. A flow diagram of study selection is presented in Fig. 1.

The selected articles were published between 2006 and 2016 and included 24 different equations (described in the Supplement). Nine studies evaluated only type 2 DM^12–14,16,23–27^, five investigated only type 1 DM^28–32^, and the remainder evaluated both or did not specify the type evaluated^15,17,19,33–38^. Among the methods used for measuring serum cystatin C, eleven studies reported the use of immunonephelometry, five used immunoturbidimetry, one reported use of both methods, one used colorimetric immunoassay, one employed colloidal gold immunoassay, and three did not report the method used. The main characteristics of the studies are shown in Table 1.

**Table 1:** Characteristics of the included studies.

Author, year, country	Sample size	Measured GFR (mL/min/1.73 m^2^)	Reference Method	Equations
MacIsaac, 2006, Australia*^†^	126	89.2 ± 3	^99m^Tc-DTPA	MacIsaac
Beauviex, 2007, France*^†^	124	56.1 ± 35	^51^Cr-EDTA	Arnal, Rule, MacIsaac, Tan
Rigalleau, 2008, France*^†^	124	56.1 ± 35	^51^Cr-EDTA	Composite (creat/cyst)
Chudleigh, 2009, UK^†^	106	104.5 ± 20	^51^Cr-EDTA	Perkins, Arnal, Rule, MacIsaac, Tan, Stevens (age), Stevens (creat)
Li H, 2010, China^†^	91	86.7 ± 27	^99m^Tc-DTPA	Perkins, MacIsaac, Rule, Stevens (creat), Ma
Cherney, 2010, Canada*	32	132.3 ± 19.7	Inulin	MacIsaac
Didangelos^b^, 2010, Greece^†^	368	72 ± 22	^51^Cr-EDTA	Stevens (age)
Iliadis, 2011, Greece^†^	448	73.4 ± 23	^51^Cr-EDTA	Perkins, Arnal, Rule, MacIsaac, Stevens (age), Stevens (creat), Tan, Grubb, Tidman, Flodin
Bevc, 2012, Slovenia^†^	113	42.9 ± 30	^51^Cr-EDTA	CKD-EPI (creat/cyst), Perkins
Inker, 2012, USA (development)*^†^	1726	68 ± 39^a^	^125^I-Iothalamate, Iohexol, ^51^Cr-EDTA	CKD-EPI (cyst)
Inker, 2012, USA (validation)*^†^	594	70 ± 41^a^	^125^I-Iothalamate, Iohexol, ^51^Cr-EDTA	CKD-EPI (cyst)
Anderson, 2012, USA*^†^	224	49 ± 21^a^	Urinary ^125^I-Iothalamate	CRIC
Chen^b^, 2013, China^†^	279	69.8 ± 21	^99m^Tc-DTPA	Stevens, Rule
Iliadis^b^, 2013, Greece*	78	106.2 ± 10	^51^Cr-EDTA	Tan
de Boer, 2014, USA*	1334	122.7 ± 21	^125^I-Iothalamate	CKD-EPI (creat/cyst), CKD-EPI (cyst)
Maahs, 2014, USA*	15	84.1 ± 15	Iohexol	CKD-EPI (creat/cyst), CKD-EPI (cyst)
Tsuda, 2014, Japan*^†^	40	68.1 ± 21	Inulin	Japanese (creat/cyst), Japanese (cyst)
Fan, 2014, USA*^†^	594^c^	69.8 ± 41^a^	^125^I-Iothalamate, Iohexol, ^51^Cr-EDTA	CKD-EPI (creat/cyst), CKD-EPI (cyst)
Mindikoglu, 2014, USA*^†^	22	83.6 ± 35^a^	^125^I-Iothalamate	CKD-EPI (creat/cyst)
Vega, 2014, Spain*^†^	31	53.48 ± 34	^99m^Tc-DTPA	Hoek
Perrin, 2015, Sweden*	104	128 (111–143)	Inulin	Schwartz (cyst), Schwartz (creat/cyst), Inker (cyst), Inker (creat/cyst), Capa, Berg
Machado^b^, 2015, Brazil^†^	84	104 ± 27	^51^Cr-EDTA	CKD-EPI (creat/cyst), CKD-EPI (cyst), CAPA
Barr, 2016, Australia^†^	216	104 (83–122)^a^	Iohexol	CKD-EPI (creat/cyst), CKD-EPI (cyst)
Kakaletsis^b^, 2016, Greece^†^	192	NR	^51^Cr-EDTA	CKD-EPI (creat/cyst), CKD-EPI (cyst)

^*^Type 1 diabetes mellitus.

^†^Type 2 diabetes mellitus.

^a^Data from overall study population, not the subgroup of diabetic patients.

^b^Published in conference annals.

^c^Same population of the validation subgroup of Inker *et al*.

NR, not reported.

Accuracy results (P10, P15, P20, P30, and P50) are shown in Table 2. Correlation ranged from 0.38 for the Berg equation to 0.92 for the CKD-EPI equation (creat/cyst) (See Supplementary Table S1).

**Table 2:** Accuracy results (P10 and P30) for the included equations.

Equation	Study	P10 (%)	P30 (%)
MacIsaac	Beauvieux	—	55
	Chudleigh	34	85
	Cherney	—	—
	Iliadis	16.5	46.4
	Li H	—	54.9
	MacIsaac	—	88
Arnal	Beauvieux	—	64
	Chudleigh	30	75
	Iliadis	17.9	45.1
Rule	Beauvieux	—	67
	Chudleigh	31	68
	Iladis	22.5	53.3
	Chen	—	—
	Li H	—	47.2
Tan	Beauvieux	—	59
	Chudleigh	34	84
	Iliadis*	—	83.3
	Iliadis	39	78.8
CKD-EPI (cyst)	Boer	—	93.2
	Inker (development)	—	89.9
	Inker (validation)	—	91.1
	Fan	—	91.8
	Machado*	—	57.1
	Maahs	—	—
	Barr	—	66.3
	Kakaletsis	—	—
CKD-EPI (creat/cyst)	Boer	—	96
	Fan	—	93.1
	Mindikoglu	—	77.3
	Machado*	—	67.9
	Bevc	—	30–72.7^†^
	Maahs	—	—
	Barr	—	86.1
	Kakaletsis	—	—
Stevens (age)	Chudleigh	29	75
	Didangelos*	33.2	72.6
	Iliadis	24	53.7
Stevens (creat)	Chudleigh	27	78
	Iliadis	24	70.5
	Li H	—	70.3
	Chen*	—	—
Perkins	Chudleigh	21	64
	Iliadis	6.6	21.2
	Li H	—	44
	Bevc	—	—
CAPA	Perrin	30	78
	Machado*	—	53.6
Grubb	Iliadis	20.9	69.1
Tidman	Iliadis	13.2	40.1
Flodin	Iliadis	16.5	43.4
Ma	Li H	—	61.5
Schwartz (cyst)	Perrin	7	53
Schwartz (creat/cyst)	Perrin	10	51
Inker (cyst)	Perrin	42	84
Inker (creat/cyst)	Perrin	45	94
Berg	Perrin	40	86
Composite (creat/cyst)	Rigalleau	—	—
Japanese (creat/cyst)	Tsuda	—	—
Japanese (cyst)	Tsuda	—	—
Hoek	Vega	—	—
CRIC	Anderson	—	83

^*^Published in conference annals. ^†^Bevc: accuracy according to stage of GFR (1–5).

Meta-analyses of accuracy could be performed for 10 equations: Arnal, CAPA, CKD-EPI (cyst), CKD-EPI (creat/cyst), MacIsaac, Perkins, Rule, Stevens (creat), Stevens (age), and Tan. We chose to pool P10-P15 accuracy values because of the small number of studies. The CKD-EPI equations were analyzed by P20 accuracy, as there was insufficient information on P10-P15. Meta-analysis of P30 accuracy was performed for all equations.

**Figure 2:**
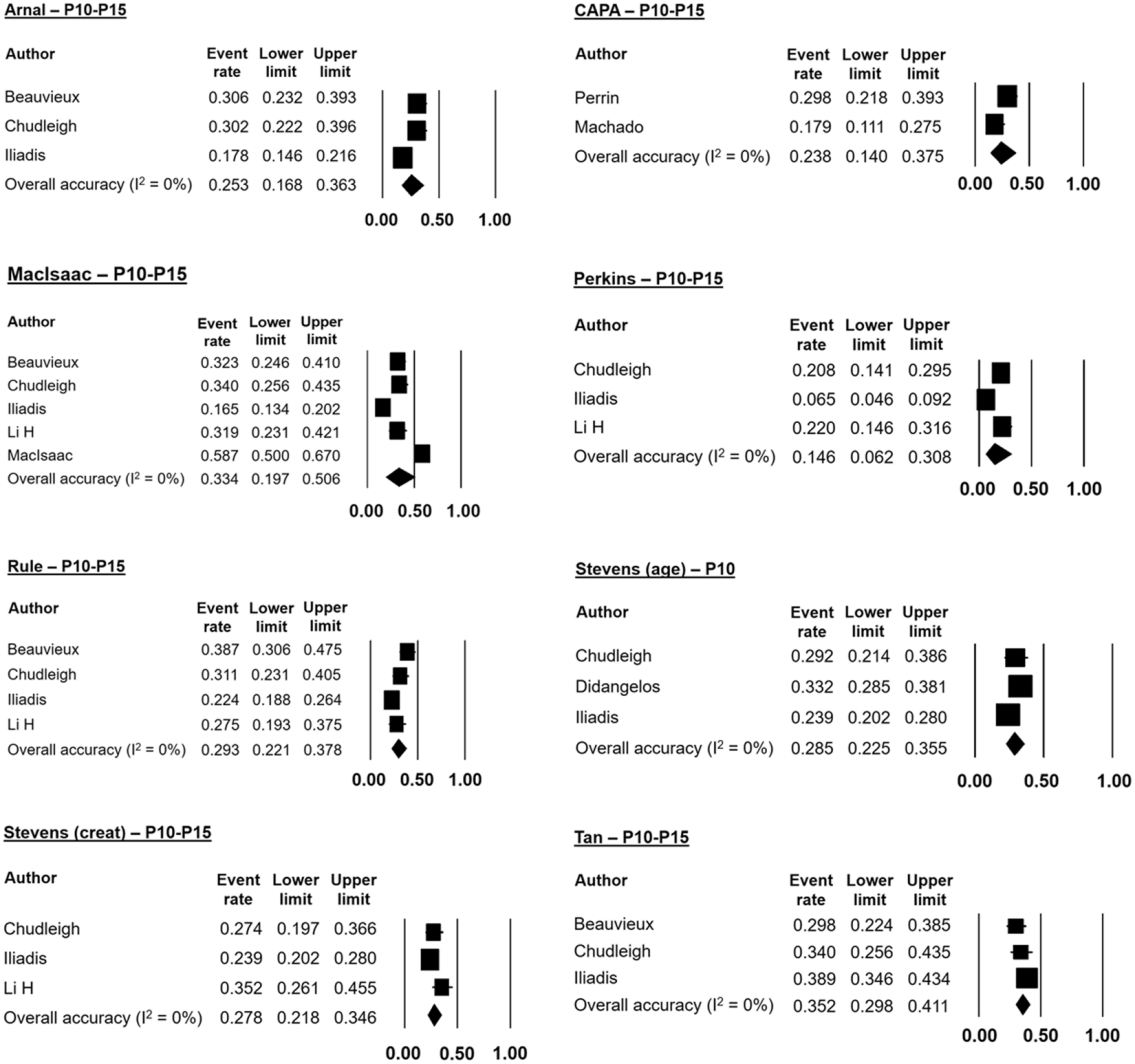
Forest plots of mean P10–15 accuracies using random effects meta-analysis.

**Figure 3:**
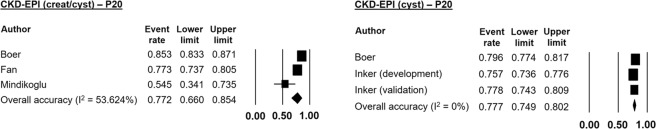
Forest plots of mean P20 accuracies using random effects meta-analysis.

**Figure 4:**
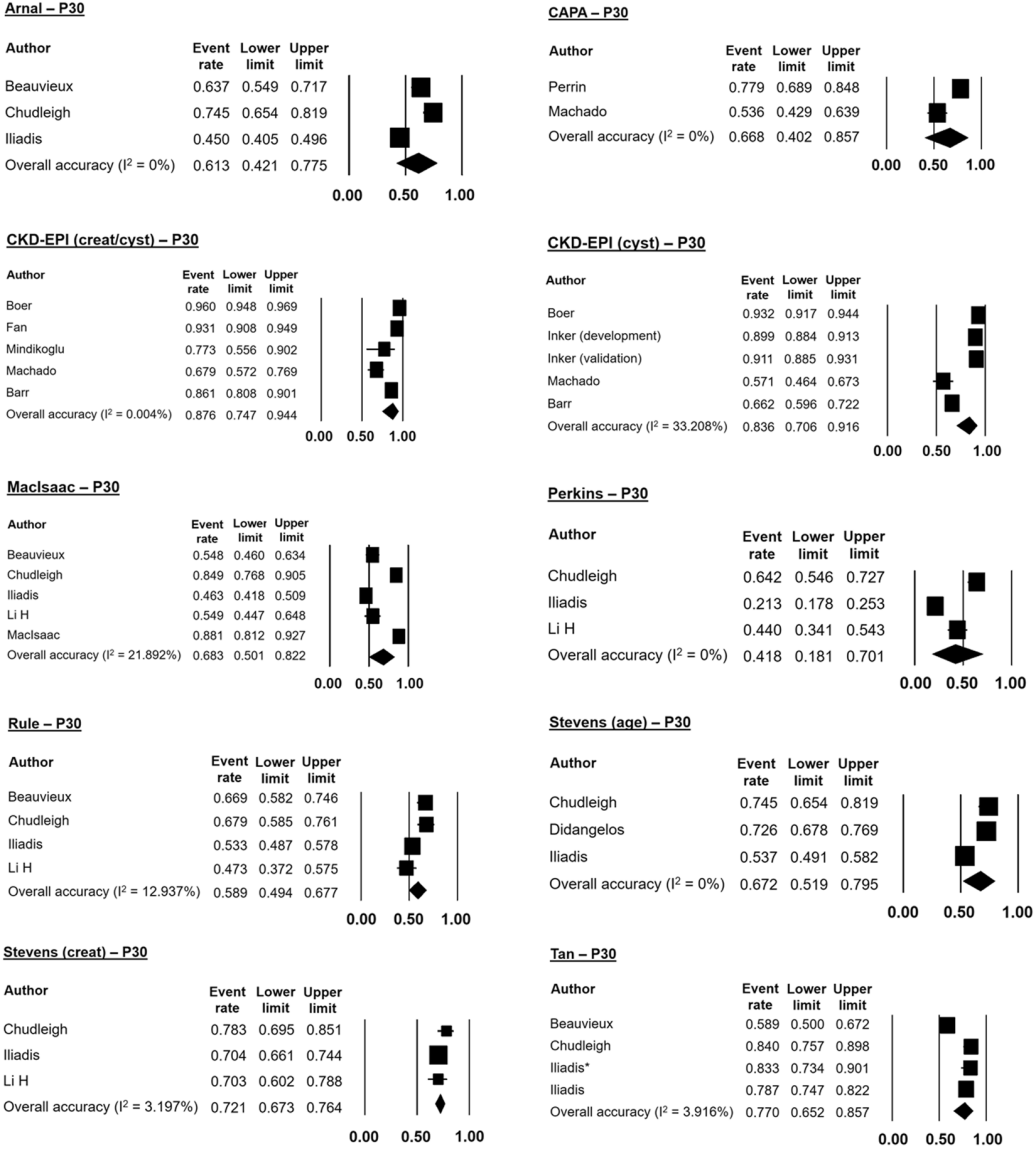
Forest plots of mean P30 accuracies using random effects meta-analysis.

Figure 2 shows the forest plots of mean P10-P15 accuracies. The Tan equation had the highest mean P10-P15 accuracy (35.2%), while the Perkins equation had the lowest (14.6%). The mean P20 accuracy of CKD-EPI equations was around 77%, whether with cystatin C alone or combined with creatinine (Fig. 3). Figure 4 illustrates the forest plots of P30 accuracy. Again, the Perkins equation had the lowest value (41.8%). The most accurate equations were CKD-EPI (creat/cyst), CKD-EPI (cyst), and Tan, with mean P30 accuracies of 87.6%, 83.6%, and 77% respectively.

Two meta-analyses had moderate heterogeneity; none had high heterogeneity. Sensitivity analysis to evaluate the effect of different reference methods for mGFR in the cases of moderate heterogeneity did not change the results. Subanalyses with studies using only traceable cystatin C were performed. The only equations with enough studies to summarize the mean accuracies were the two CKD-EPI equations: mean P20 accuracy of 81.7% for CKD-EPI (creat/cyst), and mean P30 accuracies of 92.7% and 87.6% for CKD-EPI (creat/cyst) and CKD-EPI (cyst) respectively (See Supplementary Figs S1 and S2).

Finally, the overall quality of evidence as assessed by QUADAS-2 was good (See Supplementary Fig. S3). For the “index test” and “reference standard” domains, around 60% of the studies were considered to have an unclear risk of bias because they did not describe whether eGFR and mGFR results were interpreted without knowledge of each other. However, since both the index and reference tests are objective laboratory measurements, we considered that the risk of bias could be judged as low. Ten studies (40%) did not report the interval between cystatin measurement and isotopic measurement of GFR. There was low concern regarding applicability for 21 of the 23 studies. For two studies, we had high concerns about applicability in one domain: one in the patient selection field, because all included subjects had cirrhosis, which could imply other interfering factor with renal function; and the other in the index test domain, since the equation used was validated in a cohort of individuals older than the included participants, although the authors used a conversion formula.

## Discussion

The present systematic review evaluated 23 studies that analyzed the performance of cystatin C-based eGFR equations in patients with DM across a wide range of GFRs. In order to be a useful tool to predict GFR, an equation should present satisfactory accuracy, which encompasses the concepts of bias and precision. In practical terms, accuracy is described as the proportion of cases in which GFR estimations stand within 10% (P10), 15% (P15), 20% (P20), 30% (P30), and 50% (P50) of mGFR. The mean P30 accuracies of the equations analyzed herein varied widely, from as low as 41% (with Perkins equation) to approximately 85% with the CKD-EPI equations, which offered the best performance. Either for clinical practice or for research purposes, we need GFR estimates that capture a large proportion of data that are within a meaningful boundary from target values – in this case measured GFR. Nonetheless, the boundary of ±30% of mGFR represents an unacceptably high margin of error. For example, in a patient with mGFR of 50 mL/min/1.73 m^2^, eGFR may vary from 35 to 65 mL/min/1.73 m^2^, which would represent different stages of CKD. When analyzing mean P10-P15 accuracies, a stricter comparison, proportions achieved 35% in the best scenario; that is, eGFR varied within 10 to 15% in relation to mGFR in only 35% of cases. Thus, we can consider that even the best estimates provided by such equations are inadequate representations of mGFR.

The fact that the equations were developed in different populations might have contributed to their low accuracy. For example, Rule and Stevens equations were validated in patients with CKD, mainly of hypertensive origin. However, the MacIsaac and Perkins equations, both developed specifically in patients with type 2 diabetes, also presented inadequate performance. In addition, the same equation had divergent performances in different studies and within the same study according to mGFR levels. For example, MacIsaac, Arnal, and Perkins equations had much lower P10 accuracies in the study of Iliadis *et al*.^14^ than in Chudleigh *et al*.^13^. Also in the Iliadis study, Arnal, Rule, MacIsaac, Stevens (creat) and Stevens (age) equations performed better in patients with GFR between 30–59 mL/min/1.73 m^2^ when compared to GFR between 60–89 and above 90 mL/min/1.73 m^2^. In contrast, Tan and Perkins equations had higher accuracies when GFR was above 90 mL/min/1.73 m^2^. In the study of Bevc *et al*., CKD-EPI (creat/cyst) performed better in patients with stages 4 and 5 of CKD, with a mean P30 accuracy of around 72%, compared to only 30% in patients with GFR above 90 mL/min/1.73 m^2^ ^12^.

Some factors have been associated with variations in cystatin C measurement independently of renal function. Age, body mass index (BMI), smoking, hypertension, hyperthyroidism, malignancy, increased levels of C-reactive protein, and triglycerides are associated with higher levels of cystatin C^39,40^. In most studies included in this meta-analysis, detailed information about these possible influences was unavailable; therefore, they cannot be excluded as possible factors limiting the performance of the cystatin C-based equations.

Perhaps one important potential factor interfering with the performance of the equations might have been the method used for cystatin C measurement, especially the lack of traceability of some procedures. The most widely employed methods were immunonephelometry and immunoturbidimetry. The first certified reference material for calibration of cystatin C-based assays, the so-called European Reference Material (ERM-DA471/IFCC), was only developed in 2010^41^. Seventy percent of the studies included in the present review used non-traceable methods, since most were conducted before development of the reference material. However, even after analyzing only the equations that used traceable methods, performance did not change substantially. Therefore, bias remains a major source of uncertainty, as manufacturers still have to work to improve calibration procedures^42^.

There is undeniable heterogeneity among the different equations developed for GFR estimation. Some use gender, while others do not^12,14,16,19,28,32,35–38^. Even skin color, a highly controversial issue, has been included in some formulas. Furthermore, since the equations are obtained from regression models, validation for different populations is clearly necessary, contemplating varied ranges of GFR and BMI, as well as different types of renal disease.

Currently, Kidney Disease Improving Global Outcomes (KDIGO) recommends the use of cystatin C-based equations whenever eGFR calculated with creatinine-based equations is 45–60 mL/min (borderline normality) in the absence of other evidence of kidney damage^6^. It is well known that, unlike creatinine, cystatin C is not affected by muscle mass, and, therefore, would not be influenced by gender, malnutrition, amputations, or any condition that affects creatinine^18,43^. The use of combined cystatin-creatinine equations has been gaining ground, since each analyte should theoretically balance out the limitations of the other. We had enough data to perform meta-analyses for only two combined equations: Stevens (creat) and CKD-EPI (creat/cyst). The Stevens combined equation had similarly poor results (mean P10–15 of 28%) to equations using cystatin C only. The CKD-EPI (creat/cyst) equation had the same mean P20 accuracy (77%) as CKD-EPI (cyst) equation. Therefore, in the present meta-analysis, this practice did not appear to provide any clear advantage over isolated use of cystatin C-based equations.

The growing interest in the use of cystatin C in diabetic patients originated in studies which reported encouraging predictive ability of cystatin C to anticipate cardiovascular and renal outcomes in this population – in some cases demonstrating a remarkable power to predict mortality^44–47^. Two meta-analyses in patients with DM, using receiver operating characteristic (ROC) curve data analysis, suggested that cystatin C could be a more sensitive marker for the detection of renal disease than creatinine^48^, as well as an early predictor of DKD^49^. However, these two studies employed different concepts from the present meta-analysis, using serum cystatin C alone as an indicator, not its transposition into equations for estimation of GFR. To the best of our knowledge, this was the first meta-analysis to assess the performance of cystatin C-based eGFR equations in diabetic patients by evaluating their accuracy, as recommended in the literature.

A recent mini-review evaluated the errors of estimated - creatinine or cystatin C based equations, alone or in combination - versus true GFR in patients with DM^50^. The proportion of eGFR values within ±30% (P30) ranged from 21% to 90%, highlighting the unacceptable high variability of the equations. In some series, 35% of the cases would be misclassified according to CKD stages. These findings reemphasize the perspective that P30 is an excessively high limit to validate a formula, and P10 would be a more representative test.

Although the present meta-analysis primarily focused on cystatin C-equations accuracy in diabetes, it is worthwhile to mention some aspects of the most widespread used creatinine-centered equations in this subset of patients. A recent meta-analysis compared MDRD with CKD-EPI creatinine-based equation, evaluating 48 studies (8 in diabetes), and showed that both equations underestimated mGFR, although CKD-EPI gave more accurate estimates^51^. This underestimation seems to be indeed more noticeable in patients with diabetes, even with the use of CKD-EPI equation^8,9,50^. In this sense, cystatin C-based equations would fulfill a gap. A large European multicenter study concluded that, overall, the addition of cystatin C improved precision of the combined equations compared with their creatinine equation counterparts^52^. However, once more in the diabetes scenario, cystatin C-based equations do not seem to outperform creatinine equations^26,53^.

Many studies included in this systematic review assessed correlation coefficients between estimated and measured GFR. These coefficients varied widely, from weak to relatively robust. Nevertheless, correlation is not the most appropriate statistical procedure for comparison between methods; Bland–Altman agreement analysis is the recommended exploratory method^54^. In addition, specific statistics of agreement for continuous variables - the concordance correlation coefficient (CCC), the total deviation index (TDI), and the coverage probability (CP) - have been increasingly used. CCC combines accuracy and precision, and it is scored from 0 to 1; TDI captures a huge proportion of data within a boundary for allowed differences between estimations and measurements. Regrettably, these analyses are still underused, and the studies included in the present meta-analysis did not show this kind of investigation^55^.

Some limitations of our work must be taken into account. First, there are no reliable methods to assess the risk of publication bias in systematic reviews of observational studies, and, despite our thorough literature search, the potential for such bias in the present study is unknown. Second, combining the results of P10 and P15 is debatable. Notwithstanding, we chose to put it together because of the scant data from individual studies to do separate analyzes. Third, despite the use of random-effects meta-analysis, some of the observed heterogeneity could not be explained by subgroup meta-analysis, and is likely to reflect real differences in studied populations or in the methods employed among different studies. This limits the external validity of pooled effect-size estimates. These limitations notwithstanding, we believe we were able to effectively find and summarize the current evidence on the accuracy of serum cystatin C-based equations to estimate GFR in diabetic patients.

In conclusion, the use of serum cystatin C in equations to estimate GFR, either alone or in association with serum creatinine, represent measured GFR fairly at best in DM patients, with high variability among the several proposed equations. Given the ample heterogeneity of existing equations used to estimate GFR and the preceding use of non-traceable cystatin C assays, future studies following guidelines for validation of diagnostic methods should be conducted to develop new equations with greater external validity.

## Supplementary information

Supplement
