# Supplementary material for: Performance of Cystatin C-Based Equations for Estimation of Glomerular Filtration Rate in Diabetes Patients: A Prisma-Compliant Systematic Review and Meta-Analysis
Source: Sci Rep. 2019 Feb 5;9:1418. doi: 10.1038/s41598-018-38286-9 (PMC6363744; doi:10.1038/s41598-018-38286-9)
Supplement: Supplementary file 1 — Supplement [file 41598_2018_38286_MOESM1_ESM.docx]

**PERFORMANCE OF CYSTATIN C-BASED EQUATIONS FOR ESTIMATION OF GLOMERULAR FILTRATION RATE IN DIABETES PATIENTS: A PRISMA-COMPLIANT SYSTEMATIC REVIEW AND META-ANALYSIS**

**CYSTATIN-C GFR EQUATIONS IN DIABETES: A REVIEW**

**Amanda Veiga Cheuiche^1,3^**

**Marina de Queiroz^1^**

**André Luis Ferreira Azeredo-da-Silva^2^**

**Sandra Pinho Silveiro*^1,3^**

¹Graduate Program in Medical Science: Endocrinology, Universidade Federal do Rio Grande do Sul (UFRGS), Brazil

^2^Internal Medicine Division, Hospital de Clínicas de Porto Alegre (HCPA), Brazil

^3^Endocrine Division, HCPA, Brazil

Corresponding author:

Sandra Pinho Silveiro

Serviço de Endocrinologia, HCPA

Rua Ramiro Barcelos, 2350 – Prédio 12, 4º andar

Porto Alegre, RS

E-mail: [silveirosandra@gmail.com](mailto:silveirosandra@gmail.com)

**Table of contents**

|  |  | **Page** |
| --- | --- | --- |
| Search strategy |  | 3 |
| Figure S1 | Forest plot of mean P20 accuracy for CKD-EPI (creat/cyst) with traceable cystatin C only (random effects meta-analysis). | 6 |
| Figure S2 | Forest plots of mean P30 accuracies for CKD-EPI (creat/cyst) and CKD-EPI (cyst) with traceable cystatin C only (random effects meta-analysis). | 6 |

| Figure S3 | Bar charts show assessment of quality of studies included in review and meta-analysis according to Quality Assessment of Diagnostic Accuracy Studies 2 (QUADAS-2) criteria. | 7 |  |
| --- | --- | --- | --- |
| Table S1 | Accuracy results (P10, P15, P20, P30, and P50) and correlation coefficients for the included equations. | 8 | |

**SEARCH STRATEGY**

**Search strategy used in MEDLINE database (via PubMed)**

Patient:

“Diabetes Mellitus” [Mesh] OR “diabetes mellitus” OR “diabetes”

Test:

“Cystatin C” [Mesh] OR “Post-gamma-Globulin” OR “Post-gamma-Globulin” OR “Neuroendocrine Basic Polypeptide” OR “Basic Polypeptide, Neuroendocrine” OR “Cystatin 3” OR “gamma-Trace” OR “gamma Trace”

**Search strategy used in Embase database**

Patient:

('diabetes'/exp or 'diabetes' or 'diabetes mellitus'/exp or 'diabetes mellitus')

Test:

('cystatin c'/exp or 'cystatin c') and ('glomerulus filtration rate'/exp or 'glomerulus filtration rate' or 'glomerular filtration rate')

**Description of the equations**

Cystatin C equations

- MacIsaac: (84.6/CysC)-3.2

- Arnal: 74.835/(CysC^1.333^)

- Rule: 66.8/CysC^1.30^

- Tan: (87.1/CysC)- 6.87

- CKD-EPI (cyst): 133 × (CysC/0.8)^-0.499^ × 0.996^Age^ [× 0.932 if female] if CysC <= 0.8 or 133 × (CysC/0.8)^-1.328^ × 0.996^Age^ [× 0.932 if female] if CysC >0.8

- Perkins: 100/CysC

- Stevens (age): 127.7×CysC^-1.17^×Age^-0.13^(×0.91 if female)

- CAPA: 130CysC^-1.069^ x Age^-0.117^ - 7

- Grubb: 86.49× CysC^-1.686^(×0.948 if female)

- Tidman: (100/CysC) -14

- Flodin: 79.901× CysC^-1.4389^

- Schwartz (cyst): 70.69x CysC^-0.931^

- Inker (cyst): 133x(min(CysC/(0.8);1)^-0.499^)x(max(CysC/(0.8); 1)^-1.328^)x(0.996^Age^ [x 0.932 if female)

- Berg: 91x CysC ^-1.213^

- Japanese (cyst): (104xCysC^-1.019^ x 0.996^Age^ x 0.929 if female) – 8

- Hoek: -4,32+80.35/CysC

Combined creatinine-cystatin C equations

- Stevens (creat): 77.6×(Creat/88.4)^-0.65^×CysC^-0.57^×Age^-0.20^×(0.82 if female)

- Ma: 169×(Creat/88.4)^-0.608^×CysC^-0.63^×Age^-0.157^×(0.83 if female)

- Schwartz (creat/cyst): mean (42.3 x (height (m)/Creat^0.78^ + (70.69xCysC^-0.931^))

- Inker (creat/cysC):

If male, 135x(min(Creat/(88.4x0.9);1)^-0.207^)*(max(Creat/(88.4x0.9); 1)^-0.601^)x(0.995^Age^)x(min(CysC/(0.8);1)^-0.375^)x(max(CysC/(0.8); 1)^-0.711^)

If female, 130x(min(Creat/(88.4x0.7);1)^-0.248^)x(max(Creat/(88.4x0.7); 1)^-0.601^)*(0.995^Age^)x(min(CysC/(0.8);1)^-0.375^)x(max(CysC/(0.8); 1)^-0.711^

- Composite (creat/cyst): √([66.8×(CysC)^-1.30^]×[273×(Creat)^-1.22^ ×Age^-0.299^ ×0.738 (if female)]);

- Japanese (creat/cyst): 92 x CysC^-0.575^ x Creat^-0.670^ x 0.995^Age^ (x0.784 if female)

- CKD-EPI (creat/cyst):

| ≤0.7 | |  | ≤0.8 | 130 x (Scr/0.7)^-0.248^ x (Scys/0.8)^-0.375^ x 0.995^Age^ [x 1.08 if black] |
| --- | --- | --- | --- | --- |
|  |  |  | >0.8 | 130 x (Scr/0.7)^-0.248^ x (Scys/0.8)^-0.711^ x 0.995 ^Age^  [x 1.08 if black] |
| >0.7 | |  | ≤0.8 | 130 x (Scr/0.7)^-0.601^ x (Scys/0.8)^-0.375^ x 0.995 ^Age^  [x 1.08 if black] |
|  |  |  | >0.8 | 130 x (Scr/0.7)^-0.601^ x (Scys/0.8)^-0.711^ x 0.995 ^Age^  [x 1.08 if black] |
| ≤0.9 | |  | ≤0.8 | 135 x (Scr/0.7)^-0.207^ x (Scys/0.8)^-0.375^ x 0.995 ^Age^ [x 1.08 if black] |
|  |  |  | >0.8 | 135 x (Scr/0.7)^-0.207^ x (Scys/0.8)^-0.711^ x 0.995 ^Age^ [x 1.08 if black] |
| >0.9 | |  | ≤0.8 | 135 x (Scr/0.7)^-0.601^ x (Scys/0.8)^-0.375^ x 0.995 ^Age^ [x 1.08 if black] |
|  |  |  | >0.8 | 135 x (Scr/0.7)^-0.601^ x (Scys/0.8)^-0.711^ x 0.995 ^Age^ [x 1.08 if black] |


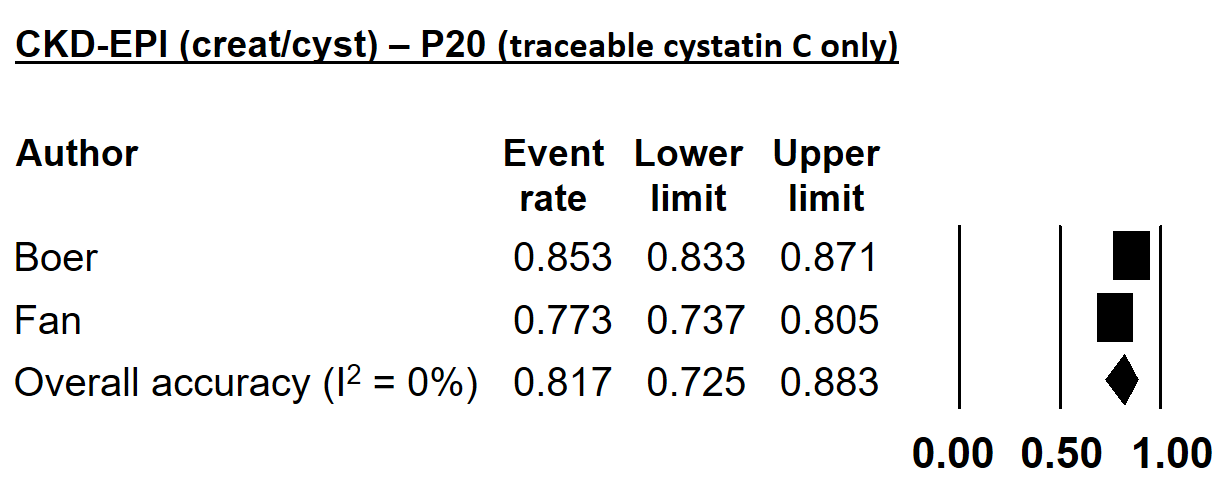


**Figure S1. Forest plot of mean P20 accuracy for CKD-EPI (creat/cyst) with traceable cystatin C only (random effects meta-analysis).**


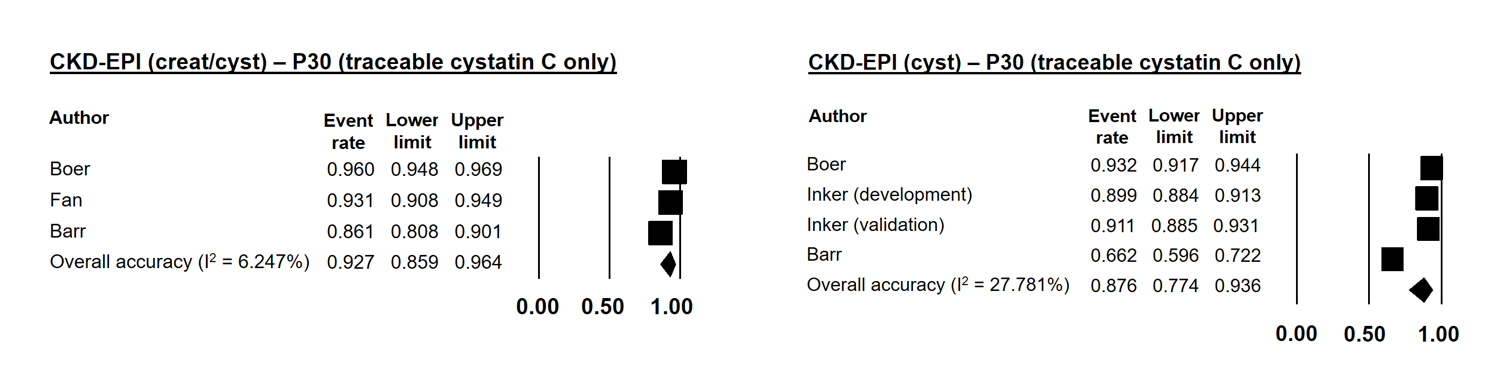


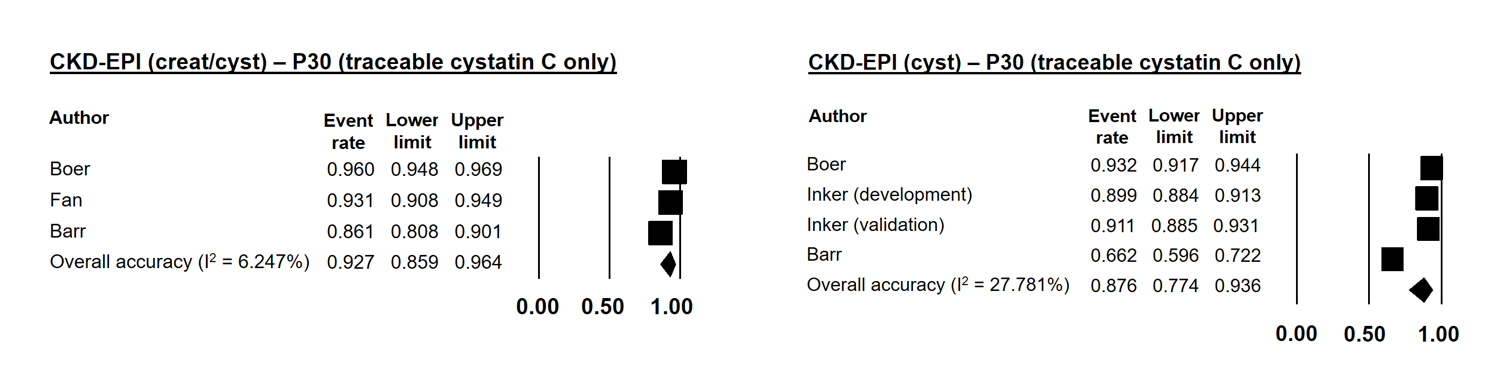


**Figure S2. Forest plots of mean P30 accuracies for CKD-EPI (creat/cyst) and CKD-EPI (cyst) with traceable cystatin C only (random effects meta-analysis).**


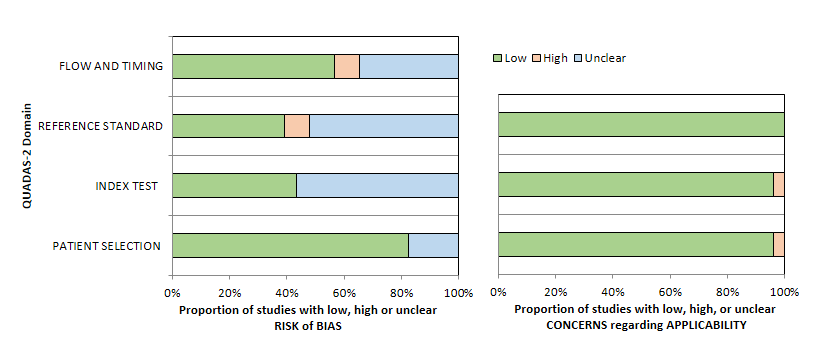


**Figure S3. Bar charts show assessment of quality of studies included in review and meta-analysis according to Quality Assessment of Diagnostic Accuracy Studies 2 (QUADAS-2) criteria.**

Table S1. Accuracy results (P10, P15, P20, P30, and P50) and correlation coefficients for the included equations.

| **Equation** | **Study** | **P10 (%)** | **P15 (%)** | **P20 (%)** | **P30 (%)** | **P50 (%)** | **Correlation coefficient** |
| --- | --- | --- | --- | --- | --- | --- | --- |
| MacIsaac | \| Beauvieux \| \| --- \| \| Chudleigh \| \| Cherney \| \| Iliadis \| \| Li H \| \| MacIsaac \| | \| -- \| \| --- \| \| 34 \| \| -- \| \| 16.5 \| \| -- \| \| -- \| | \| 32 \| \| --- \| \| -- \| \| -- \| \| -- \| \| 31.9 \| \| 59 \| | \| -- \| \| --- \| \| -- \| \| -- \| \| -- \| \| -- \| \| -- \| | \| 55 \| \| --- \| \| 85 \| \| -- \| \| 46.4 \| \| 54.9 \| \| 88 \| | \| 72 \| \| --- \| \| -- \| \| -- \| \| -- \| \| 72.5 \| \| 98 \| | \| 0.82^a^ \|  \| \| --- \| --- \| \| -- \|  \| \| 0.73^b^ \|  \| \| -- \|  \| \| -- \|  \| \| 0.87^a^ \|  \| |
| Arnal | \| Beauvieux \| \| --- \| \| Chudleigh \| \| Iliadis \| | \| -- \| \| --- \| \| 30 \| \| 17.9 \| | \| 31 \| \| --- \| \| -- \| \| -- \| | \| -- \| \| --- \| \| -- \| \| -- \| | \| 64 \| \| --- \| \| 75 \| \| 45.1 \| | \| 87 \| \| --- \| \| -- \| \| -- \| | \| 0.76^a^ \| \| --- \| \| -- \| \| -- \| |
| Rule | \| Beauvieux \| \| --- \| \| Chudleigh \| \| Iladis \| \| Chen \| \| Li H \| | \| -- \| \| --- \| \| 31 \| \| 22.5 \| \| -- \| \| -- \| | \| 39 \| \| --- \| \| -- \| \| -- \| \| -- \| \| 27.5 \| | \| -- \| \| --- \| \| -- \| \| -- \| \| -- \| \| -- \| | \| 67 \| \| --- \| \| 68 \| \| 53.3 \| \| -- \| \| 47.2 \| | \| 89 \| \| --- \| \| -- \| \| -- \| \| 88.9 \| \| 75.8 \| | \| 0.81^a^ \| \| --- \| \| -- \| \| -- \| \| -- \| \| -- \| |
| Tan | \| Beauvieux \| \| --- \| \| Chudleigh \| \| Iliadis* \| \| Iliadis \| | \| -- \| \| --- \| \| 34 \| \| -- \| \| 39 \| | \| 30 \| \| --- \| \| -- \| \| -- \| \| -- \| | \| -- \| \| --- \| \| -- \| \| -- \| \| -- \| | \| 59 \| \| --- \| \| 84 \| \| 83.3 \| \| 78.8 \| | \| 77 \| \| --- \| \| -- \| \| -- \| \| -- \| | \| 0.82^a^ \| \| --- \| \| -- \| \| -- \| \| -- \| |
| CKD-EPI (cyst) | \| \| Boer \| \| --- \| \| Inker (d) \| \| Inker (v) \| \| Fan \| \| Machado* \| \| Maahs \| \| Barr \| \| Kakaletsis \| \| \| --- \| --- \| --- \| --- \| --- \| --- \| --- \| --- \| --- \| | \| -- \|  \| \| --- \| --- \| \| -- \|  \| \| -- \|  \| \| -- \|  \| \| -- \|  \| \| -- \|  \| \| -- \|   -- | \| -- \| \| \| --- \| --- \| \| -- \| \| \| -- \| \| \| -- \| \| \| 16.7 \| \| \| -- \| \| \| -- \|   -- | \| 79.6 \| \| --- \| \| 75.7 \| \| 77.8 \| \| 75.3 \| \| -- \| \| -- \| \| -- \|   -- | \| 93.2 \| \| --- \| \| 89.9 \| \| 91.1 \| \| 91.8 \| \| 57.1 \| \| -- \| \| 66.3 \|   -- | \| -- \|  \| \| --- \| --- \| \| -- \|  \| \| -- \|  \| \| -- \|  \| \| -- \|  \| \| -- \|  \| \| -- \|   -- | \| 0.42^b^ \| \| --- \| \| -- \| \| -- \| \| -- \| \| 0.63^a^ \| \| 0.68^a^ \| \| -- \|   0.69^a^ |
| CKD-EPI (creat/cyst) | \| \| Boer \| \| --- \| \| Fan \| \| Mindikoglu \| \| Machado* \| \| Bevc \| \| Maahs \| \| Barr \| \| Kakaletsis \| \| \| --- \| --- \| --- \| --- \| --- \| --- \| --- \| --- \| --- \| | \| -- \|  \| \| --- \| --- \| \| -- \|  \| \| -- \|  \| \| -- \|  \| \| -- \| \| -- \| \| -- \|   -- | \| -- \| \| --- \| \| -- \| \| -- \| \| 33 \| \| -- \| \| -- \| \| -- \|   -- | \| 85.3 \| \| --- \| \| 77.3 \| \| 54.5 \| \| -- \| \| -- \| \| -- \| \| -- \|   -- | \| 96 \|  \| \| \| --- \| --- \| --- \| \| 93.1 \|  \| \| \| 77.3 \|  \| \| \| 67.9 \|  \| \| \| 30-72.7^†^ 7* \| \|  \| \| \| -- \| \|  \| \| \| 86.1 \| \|  \| \|   -- | \| -- \| \| --- \| \| -- \| \| -- \| \| -- \| \| -- \| \| -- \| \| -- \|   -- | \| 0.48^b^ \|  \| \| --- \| --- \| \| -- \|  \| \| -- \|  \| \| 0.63^a^ \|  \| \| 0.92^a^ \| \| 0.68^a^ \| \| -- \|   0.78^a^ |
| Stevens (age) | \| Chudleigh \| \| --- \| \| Didangelos* \| \| Iliadis \| | \| 29 \| \| --- \| \| 33.2 \| \| 24 \| | \| -- \| \| --- \| \| -- \| \| -- \| | \| -- \| \| --- \| \| -- \| \| -- \| | \| 75 \| \| --- \| \| 72.6 \| \| 53.7 \| | \| -- \| \| --- \| \| -- \| \| -- \| | \| -- \| \| --- \| \| -- \| \| -- \| |
| Stevens (creat) | \| Chudleigh \| \| --- \| \| Iliadis \| \| Li H \| \| Chen* \| | \| 27 \| \| --- \| \| 24 \| \| -- \| \| -- \| | \| -- \| \| --- \| \| -- \| \| 35.1 \| \| -- \| | \| -- \| \| --- \| \| -- \| \| -- \| \| -- \| | \| 78 \| \| --- \| \| 70.5 \| \| 70.3 \| \| -- \| | \| -- \| \| --- \| \| -- \| \| 86.8 \| \| 89.6 \| | \| -- \|  \| \| --- \| --- \| \| -- \|  \| \| -- \|  \| \| -- \|  \| |
| Perkins | \| Chudleigh \|  \| \| --- \| --- \| \| Iliadis \|  \| \| Li H \|  \| \| Bevc \|  \| | \| 21 \| \| --- \| \| 6.6 \| \| -- \| \| -- \| | \| -- \| \| --- \| \| -- \| \| 22 \| \| -- \| | \| -- \|  \| \| --- \| --- \| \| -- \|  \| \| -- \|  \| \| -- \|  \| | \| 64 \| \| --- \| \| 21.2 \| \| 44 \| \| -- \| | \| -- \| \| --- \| \| -- \| \| 60.4 \| \| -- \| | \| -- \| -- \| \| --- \| --- \| \| -- \| -- \| \| -- \| -- \| \| 0.88^a^ \|  \| |
| CAPA | \| Perrin \| \| --- \| \| Machado* \| | \| 30 \| \| --- \| \| -- \| | \| -- \| \| --- \| \| 17.9 \| | \| -- \| \| --- \| \| -- \| | \| 78 \| \| --- \| \| 53.6 \| | \| -- \| \| --- \| \| -- \| | \| 0.44^a^ \| \| --- \| \| 0.61^a^ \| |
| Grubb | Iliadis | 20.9 | -- | -- | 69.1 | -- | -- |
| Tidman | Iliadis | 13.2 | -- | -- | 40.1 | -- | -- |
| Flodin | Iliadis | 16.5 | -- | -- | 43.4 | -- | -- |
| Ma | Li H | -- | 36.3 | -- | 61.5 | 81.3 | -- |
| Schwartz (cyst) | Perrin | 7 | -- | -- | 53 | -- | 0.39^a^ |
| Schwartz (creat/cyst) | Perrin | 10 | -- | -- | 51 | -- | 0.54^a^ |
| Inker (cyst) | Perrin | 42 | -- | -- | 84 | -- | 0.49^a^ |
| Inker (creat/cyst) | Perrin | 45 | -- | -- | 94 | -- | 0.61^a^ |
| Berg | Perrin | 40 | -- | -- | 86 | -- | 0.38^a^ |
| Composite (creat/cyst) | Rigalleau | -- | -- | -- | -- | -- | 0.90^a^ |
| Japanese (creat/cyst) | Tsuda | -- | -- | -- | -- | -- | 0.71^c^ |
| Japanese (cyst) | Tsuda | -- | -- | -- | -- | -- | 0.58^c^ |
| Hoek | Vega | -- | -- | -- | -- | -- | 0.90^c^ |
| CRIC | Anderson | -- | -- | -- | 83 | -- | -- |

*Published in conference annals. † Bevc: accuracy according to stage of GFR (1-5). ^a^ Pearson correlation coefficient. ^b^ Spearman correlation coefficient. ^c^ Intraclass correlation coefficient.
